# The non glycanated endocan polypeptide slows tumor growth by inducing stromal inflammatory reaction

**DOI:** 10.18632/oncotarget.2614

**Published:** 2014-10-21

**Authors:** Hanane Yassine, Nathalie De Freitas Caires, Florence Depontieu, Arnaud Scherpereel, Ali Awad, Anne Tsicopoulos, Christophe Leboeuf, Anne Janin, Catherine Duez, Bogdan Grigoriu, Philippe Lassalle

**Affiliations:** ^1^ Institut Pasteur de Lille, Center for Infection and Immunity of Lille, Lille, France; ^2^ Univ Lille Nord de France, Lille, France; ^3^ CNRS, UMR 8204, Lille, France; ^4^ Institut National de la Santé et de la Recherche Médicale, Lille, France; ^5^ Lunginnov, Lille, France; ^6^ CHRU Lille, Hôpital Calmette, Lille, France; ^7^ Institut National de la Santé et de la Recherche Médicale, Paris, France; ^8^ Regional Institute of Oncology, Iasi, Romania; ^9^ University of Medicine and Pharmacy “Gr.T.Popa” Iasi, Iasi, Romania

**Keywords:** Mouse, Endocan, Glycosaminoglycan, Tumor, Leukocytes

## Abstract

Endocan expression is increasingly studied in various human cancers. Experimental evidence showed that human endocan, through its glycan chain, is implicated in various processes of tumor growth. We functionally characterize mouse endocan which is also a chondroitin sulfate proteoglycan but much less glycanated than human endocan. Distant domains from the O-glycanation site, located within exons 1 and 2 determine the glycanation pattern of endocan. In opposite to the human homologue, overexpression of mouse endocan in HT-29 cells delayed the tumor appearance and reduced the tumor growth rate. This tumor growth inhibition is supported by non glycanated form of mouse endocan. Non glycanated human endocan overexpressed in HT-29, A549 or K1000 cells also exhibited an anti-tumor effect. Moreover, systemic delivery of non glycanated human endocan also results in HT-29 tumor growth delay. *In vitro*, endocan polypeptide did not affect HT-29 cell proliferation, nor cell viability. In tumor tissue sections, a stromal inflammatory reaction was observed only in tumors overexpressing endocan polypeptide, and depletion of CD122+ cells was able to delete partially the anti-tumor effect of endocan polypeptide. These results reveal a novel pathway for endocan in the control of tumor growth, which involves inflammatory cells of the innate immunity.

## INTRODUCTION

Proteoglycans (PGs) are ubiquitous molecules composed of a core protein to which chains of glycosaminoglycans are attached [1]. They are involved in regulating tumor cell growth, survival, adhesion, metastasis and angiogenesis [1, 2] and are produced by both tumor and stromal cells such as fibroblast and endothelial cells [3]. Growing evidence suggests that PGs are potential therapeutic targets for cancer [1-4].

Endocan, previously called endothelial specific molecule-1 (ESM-1), is a circulating dermatan sulfate proteoglycan secreted specifically by endothelial cells [5, 6]. It is up-regulated by pro-angiogenic factors like VEGF and FGF-2 [6] and an overexpression was found in specialized endothelial tip cells in various mouse models of retinal neovascularization, and recently in human invasive bladder cancer [7-10]. Endocan also exerts a role in vascular sprouting and stimulates the VEGF induced migration of endothelial cells [7, 11, 12]. Endocan is also one of the 6 genes specifically overexpressed during angiogenic switch [13] while low expression of endocan appears to characterize dormant tumors [14].

In mice models of tumor xenografts, overexpression of human endocan induces tumor growth of otherwise non tumorigenic HEK293 cells and accelerate tumor growth of the tumorigenic HT-29 cells [15]. The glycan moiety of endocan plays an important role in this process. It binds to and promotes the mitogenic activity of hepatocyte growth factor (HGF)/scatter factor (SF) on HEK293 cells [16]. Endocan has also been shown to bind to the leukocyte integrin LFA-1 *in vitro*, and to inhibit LFA-1/ICAM-1 interaction [17] with putative implications in blocking intratumoral leucocyte recruitment.

In human tumors, endocan is overexpressed by endothelial cells of various cancers like non-small lung cancer (NSCLC), hepatocarcima (HCC), and bladder cancer [7, 18-21]. Multiple studies identified endocan expression as part of molecular signatures defining a poor prognosis in NSCLC, breast cancer, glioblastoma and HCC [18, 22-24].

To better understand the role of this molecule in the development of experimental models of solid tumors, we investigated its mouse ortholog. Mouse endocan cDNA predicts a polypeptide of 184 amino acids, sharing 75% identity with human homolog, and has an identical structural organisation: (1) an NH2-terminal hydrophobic signal peptide (2) a cysteine rich region (18 cysteines out of the first 110 amino acids) (3) a functionally defined F-rich domain ^113^FPFFQY^118^ (4) and a unique O-glycanation site on serine 138 (SGDG).

We show here that (i) mouse endocan is also a proteoglycan containing a single chondroitin sulfate chain; (ii) the secreted mouse endocan is variably glycanated; (iii) mouse endocan inhibits spontaneous tumor growth; (iv) non glycanated mouse and human endocan act similarly in delaying growth of various experimental tumors; (v) stromal inflammatory reaction is observed in tumors overexpressing endocan polypeptide; (vi) the anti-tumor property of endocan polypeptide requires the presence of CD122+ cells.

## RESULTS

### Mouse endocan is a chondroitin sulfate proteoglycan and less glycanated than its human counterpart

**Figure 1:**
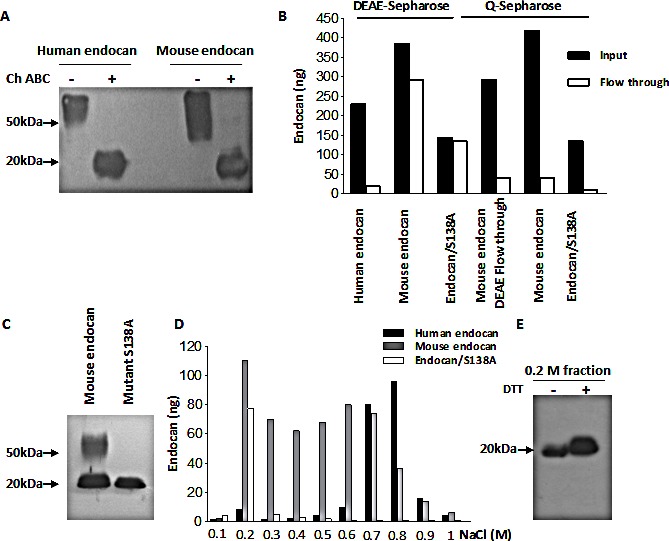
Characterization of recombinant mouse endocan (A) DEAE-Sepharose-bound eluted human and mouse endocan. Part of the samples was digested overnight with chondroitinase ABC (+). Samples were analyzed under non reducing condition. (B) DEAE- or Q sepharose elution profiles of human and mouse endocan. The amounts of the input (black boxes) and the flow through (white boxes) were measured by ELISA. (C) Western blot of crude supernatants of mouse wild type and mutant S138A endocan under non reducing condition. (D) Q-sepharose profiles of human, mouse and mutant mouse endocan/S138A. Supernatants were passed through Q-sepharose and bound endocan was eluted with NaCl gradient from 0.1 to 1 M. (E) Western blot of 0.2 M NaCl Q-sepharose-eluted fraction under non reducing (−) and reducing condition (+).

The supernatants of HEK293 cells overexpressing recombinant human and mouse endocan were passed through the DEAE-sepharose, a weak anion exchange resin which binds human endocan (20). This was also observed for mouse endocan. The DEAE-bound mouse endocan was detected on western Blot at 50 kDa. After enzymatic digestion by chondroitinase ABC, the apparent molecular weight of mouse endocan switched to 20 kDa, indicating that the DEAE-bound mouse endocan was also glycanated (Fig. 1A).

However, contrary to the human homologue, a high proportion of mouse endocan did not bind to the DEAE-sepharose (Fig. 1B). Western blotting of crude supernatants of mouse endocan producing cells revealed two bands at 50 kDa and 20 kDa (Fig. 1C) corresponding to the glycanated, DEAE-bound fraction and the protein core respectively. The second band, is similar to the non glycanable mouse endocan/S138A (Fig. 1C).

The mouse endocan DEAE flow-through, the mouse endocan as well as mutant mouse endocan/S138A bind to Q-sepharose, a strong anion exchange resin (Fig. 1B). We performed a Q-sepharose step gradient elution of mouse endocan in order to separate the glycanated and non glycanated forms. Human and mouse endocan/S138A served as controls and were eluted at 0.7-0.8 M NaCl and 0.2 M NaCl respectively. Mouse endocan was eluted in two major peaks: one at 0.2 M and a second at 0.6-0.7 M NaCl (Fig. 1D). Western blot revealed that the 0.2 M NaCl fraction corresponded to a non glycanated form of mouse endocan with an apparent molecular weight of 20 kDa (Fig. 1E).

Next, we compared the glycanation level of mouse endocan produced by transient transfection in 3 rodent (B16F10, NIH3T3, CHO-DG44) and 3 human (HEK293, HT-29 and A549) cell lines. After immuno-precipitation western blotting of endocan indicated that whatever the cell type used, mouse endocan has a significant non glycanated fraction (data not shown) suggesting that attachment of glycanic chain is independent of cell type.

### Endogenous mouse endocan shows also incomplete glycanation

**Figure 2:**
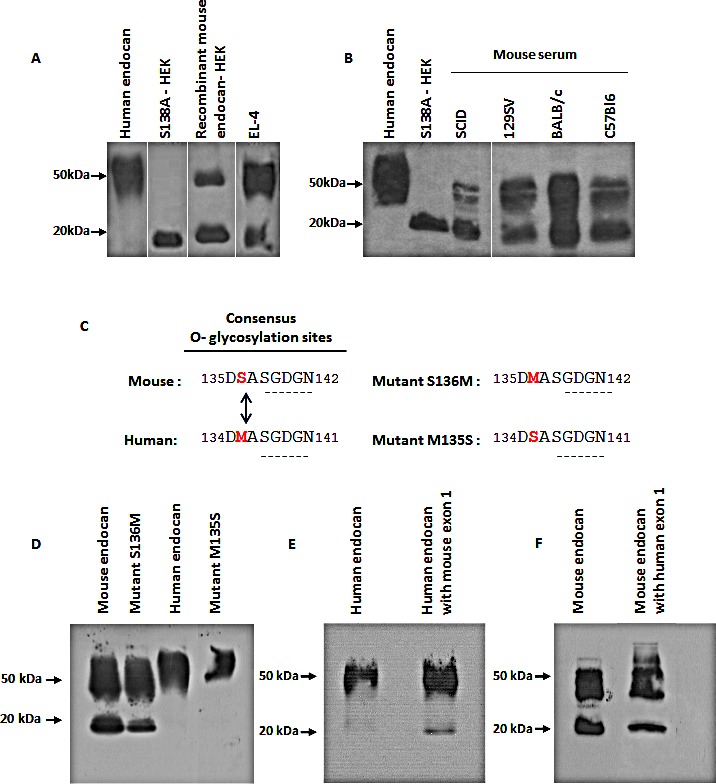

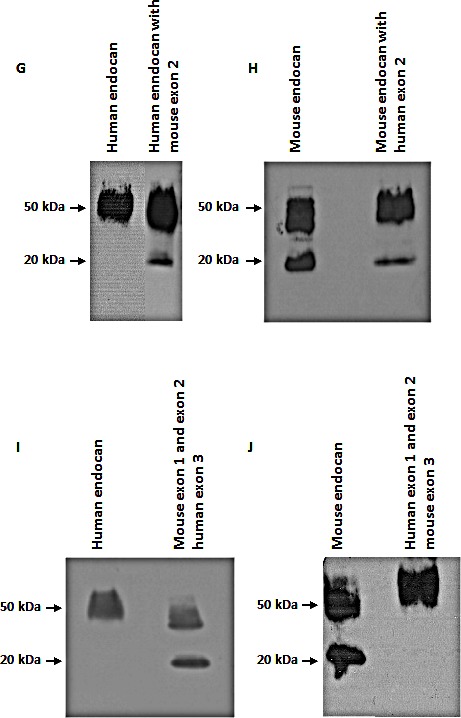
Characterization of mouse endocan (A) Immunoprecipitation and western blot of HEK293 cells overexpressing recombinant mouse endocan, EL-4 cell supernatant, (B) and sera from 4 mouse strains (SCID, 129Sv, Balb/C and C57Bl6). Samples were analyzed under non reducing condition. (C) Sequence comparison between human and mouse endocan. (D) Immunoprecipitation and western blot of HEK293 cell supernatants overexpressing wild type mouse endocan, mutant S136M, and wild type human endocan, mutant M135S, (E, F) chimaeras constructions of exon 1, (G,H) chimaeras constructions of exon 2 and (I, J) chimaeras constructions of exon 3.

To verify if natural mouse endocan also exhibit a non glycanated fraction, we first screened several mouse cell lines and found that EL-4, a T-lymphoblastic cell line, spontaneously produces endocan. Immunoprecipitation of EL-4 supernatants revealed 2 forms of endocan with molecular wiegths of 50 kDa and 20 kDa, indicating that in this cell line, endocan is spontaneously produced in both glycanated and non glycanated forms in proportions similar to the recombinant mouse endocan (Fig. 2A).

To confirm the particular glycanation profile of mouse endocan, sera of 4 mouse strains (SCID, 129Sv, Balb/C and C57Bl6) were immunoprecipitated. Similarly to EL-4 supernatants, murine serum endocan appeared in both sizes of 50 kDa and 20 kDa corresponding to the glycanated and non glycanated forms (Fig. 2B). These results confirm that the endogenous mouse endocan is also produced in both glycanated and non glycanated forms.

### Involvement of core protein sequence encoded by exons 1 and 2 in the glycanation pattern of endocan

To explain why endocan is variably glycanated, we compared the protein sequences of human and mouse endocan. Within the consensus O-glycanation sequence, only one single amino acid difference was found with the Serine 136 from murine ortholog being replaced by a Methionine in the human sequence (Fig. 2C). We therefore engineered recombinant sequences where the Ser 136 was replaced by a Met in the mouse molecule, and inversely, the Met was replaced by a Ser in the human sequence. After transient transfection in HEK293 cells, culture supernatants were immunoprecipitated using MEP14 antibody and blotted. These mutations did not change the glycanation profiles of both human and mouse endocan (Fig. 2D).

To explore which protein domain(s) govern the glycanation status, we took advantage that mouse and human endocan are both organized in 3 exons with coding sequences highly similar in size. Recombinant chimaeras were engineered and exon 1, exon 2 or exon 3 were exchanged between species. Results shows that substitution of the human by mouse sequence of exon 1 partially altered the glycanation status (Fig. 2E) while the substitution of mouse by human exon 1 sequence, was able to improve the glycanation (Fig. 2F). Similar observations were obtained after exon two substitutions (Fig. 2G, H). No change in glycanation profile of either human or mouse endocan (Fig. 2I, J) was obtained after modification of the third exon.

### Mouse endocan delays tumor growth

**Figure 3:**
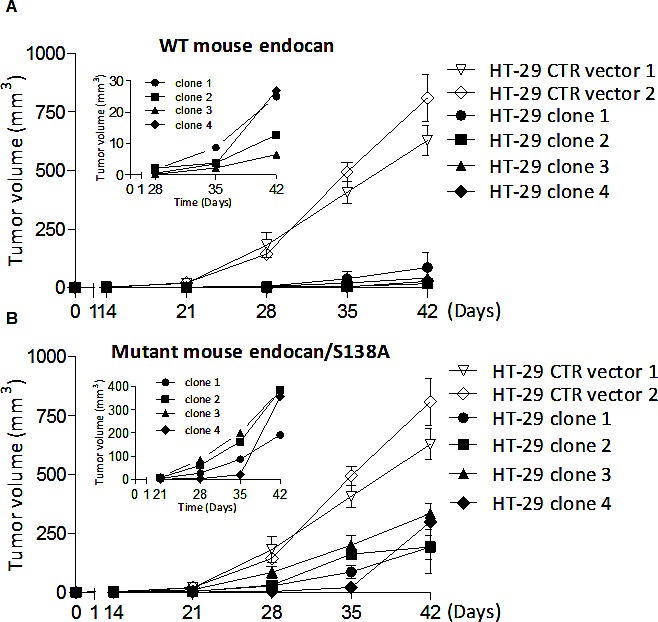

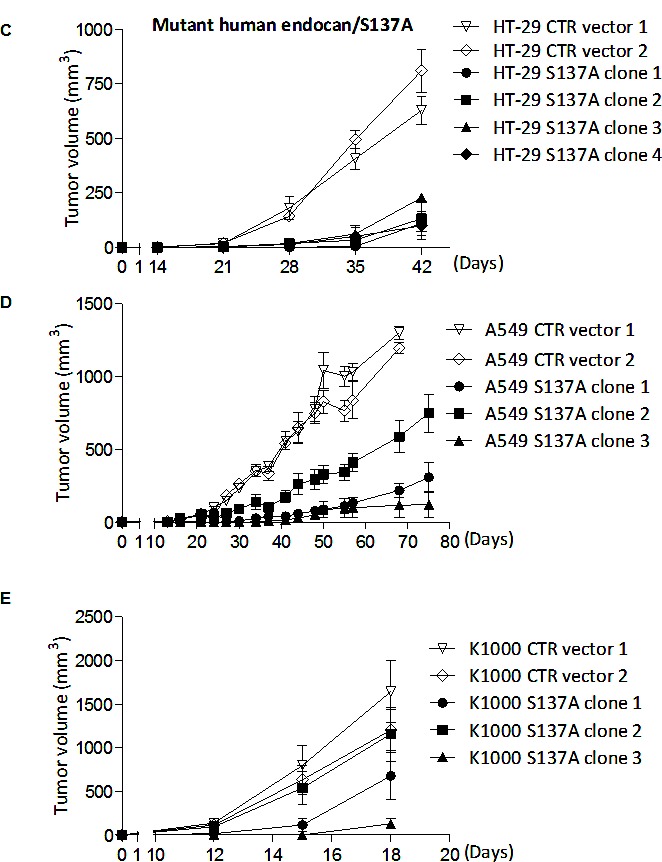
Tumor growth effect of HT-29 cells overexpressing mouse and non glycanated human endocan (A) HT-29 cells overexpressing mouse wild type (WT) endocan (black box), or control vector (white box) (*n*=4 per group, mean ± SEM of 2 experiments). The four 4 distinct clones secreted similar levels of mouse endocan (22; 20; 28; 33 ng/24h/10^6^ cells respectively). (B) Tumor growth kinetics of HT-29 cells overexpressing mutant mouse endocan/S138A. Kinetics of tumor growth of (C) HT-29 cells (D) A549 cells and (E) K1000 cells overexpressing mutant human endocan/S137A (black box) or control vector (white box) (*n*=4 per group; mean ± SEM of 2 experiments).

Since overexpression of human endocan promotes tumor formation and increases tumor growth in SCID mice [15], we investigated if mouse endocan exhibited such a tumorigenic activity. Stably transfected mouse-endocan producing HEK cells do not exibit tumoral potential when injected s.c. in SCID mice (not shown) while tumorigenic HT-29 cells exhibited a delayed subcutaneous growth in SCID mice after stable transfection with an mouse endocan producing vector and this independently of clonal selection (Fig. 3A).

### Non glycanated endocan inhibits tumor growth

Since the glycan moiety of endocan is required for tumor growth, we wondered if the non glycanated mouse endocan may have opposite effect. All four clones of the HT-29 cell line stably expressing-mouse endocan/S138A at similar levels (22; 28; 20; 24 ng/24h/10^6^) developed at a slower rate than the control HT-29 (Fig. 3B). Similarly, 4 distinct HT-29 cell clones stably expressing the non glycanated human endocan/S137A (28.5; 30.5; 31.2; 34.2 ng/24h/10^6^) showed a slower tumor growth when injected subcutaneously into SCID mice (Fig. 3C). This property was also present in two other tumorigenic cell lines (A549 and K1000) where tumor growth was delayed and slower after subcutaneous injection (Fig. 3D, E).

### Systemic administration of non glycanated endocan delayed tumor growth

**Figure 4:**
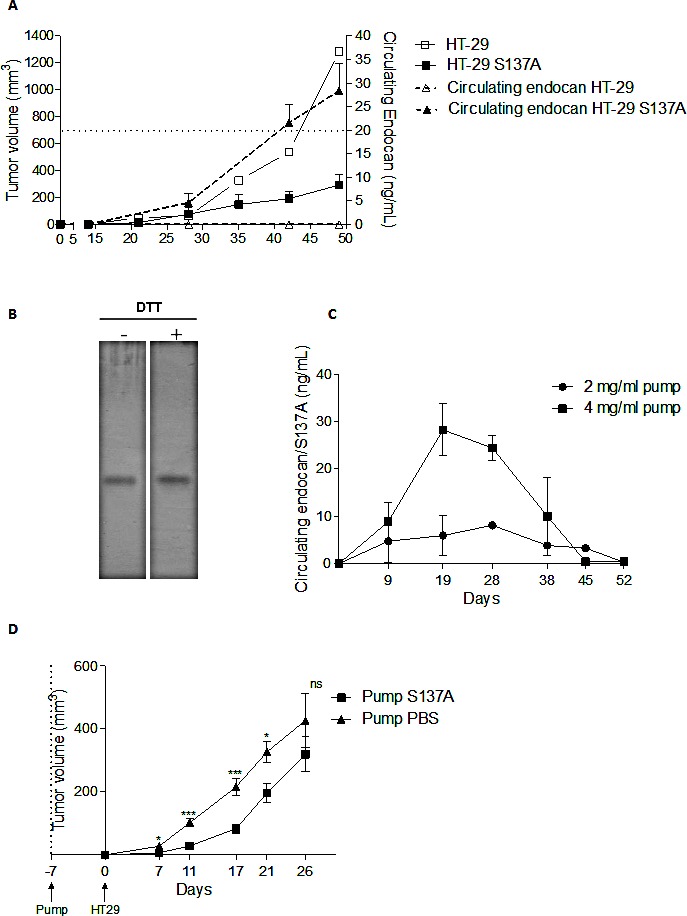
Tumor growth effect of non glycanated human endocan systemically delivered (A) Blood endocan monitoring of HT-29 tumors or overexpressing human endocan/S137A. (B) Silver-stain gel of purified human endocan/S137A. (C) Pharmacokinetics of endocan/S137A delivered by a 4-weeks duration osmotic pumps. (D) Tumor growth of HT-29 treated by osmotic pumps containing either 4 mg/mL endocan/S137A or PBS. *: p<0.05, **: p<0.01, ***: p<0.001.

As endocan is a freely circulating molecule, in mice bearing HT-29 tumors overexpressing endocan/S137A human endocan, circulating levels of this molecule increased in parallel to the tumor size (Fig. 4A).

We investigated if circulating endocan could influence the tumor growth, by systemic administration of human endocan/S137A in mice bearing HT-29 tumors. Subcutaneous ALZET osmotic pumps loaded with purified recombinant human endocan/S137A (purity > 95% - Fig. 4B) was inserted subcutaneously into SCID mice and blood levels were followed. An input of 4 mg/mL human endocan/S137A was necessary to maintain a sustained endocan blood level of 20 ng/mL for 1 month (Fig. 4C).

Osmotic pumps were inserted one week before HT-29 cell injection. This resulted in a sustained growth delay of resultant tumors for 21th day after the tumor implantation, which corresponds to the 28 days duration of osmotic pumps (Fig. 4D).

Taken together these results demonstrate that the polypeptide core of endocan is able by itself to delay the tumor growth.

### Non glycanated endocan does not influence cell proliferation and viability

**Figure 5:**
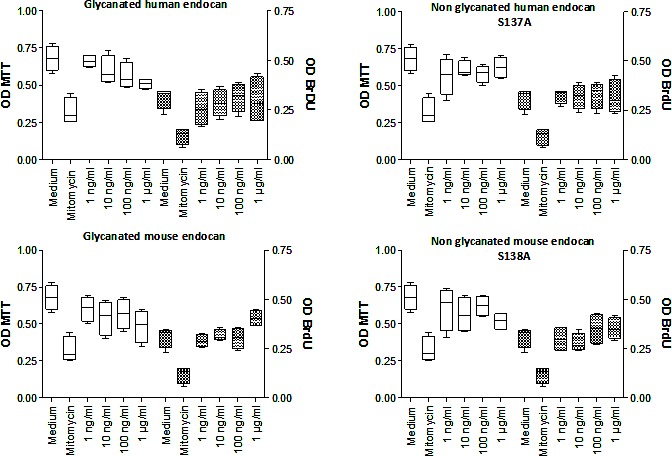
Cell proliferation effect of non glycanated human and mouse endocan Each panel represent the MTT OD (White box) and the BrDU OD (cross hatch box) after 24 hours of HT-29 culture with medium, Mytomycine (100μg/ml), or various concentrations of endocan ranges from 1 ng/ml to 1000ng/ml. Glycanated (left graphics) and non glycanated (right graphics) human and mouse endocan were tested. The bottom, median and top lines of the box mark the 25^th^, 50^th^, 75^th^ percentiles, respectively. The vertical line shows the range of values comprised between the 5^th^ and the 95^th^ percentiles.

To explore the mechanism of tumor growth inhibition by the non glycanated endocan, we tested its influence on cell proliferation and viability. Cellular proliferation assessed by BrDU incorporation was not influenced whatever the dose of human or mouse endocan was used (from 1 to 1000 ng/mL) (Fig. 5). No difference was observed between glycanated and non glycanated endocan, of either human or mouse origin. The viability of HT-29 was also not affected by glycanated or non glycanated human or mouse endocan (Fig. 5). These results suggest that the endocan polypeptide does not directly influence tumor cell proliferation.

### CD122+ leukocytes are involved in delaying tumor growth by endocan polypeptide

**Figure 6:**
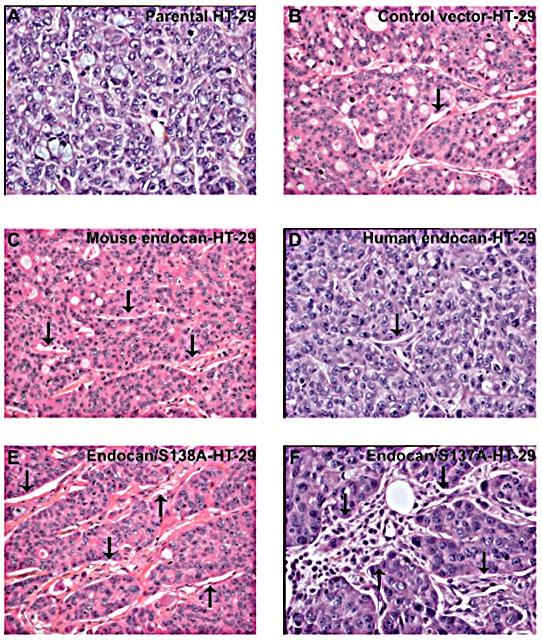
Pathological analysis of HT-29 tumors overexpressing endocan Histological section of (A) HT-29 tumor transfected with control vector, (B) parental non transfected HT-29 tumor, (C) HT-29 tumor transfected with mouse endocan, (D) HT-29 tumor transfected with human endocan, (E) HT-29 tumor transfected with endocan/S138A, (F) HT-29 tumor transfected with endocan/S137A. For all panels: Hematoxylin-eosin staining x400 magnification.

Pathological analysis of mice harbouring HT-29 tumors overexpressing mouse or human endocan showed only a local tumor development with no metastases whatever the tumor type. Microscopically, the percentage of necrotic areas did not differ between different tumor types. A more abundant stroma and a significant pan-leukocytic infiltration at the tumor periphery, and within the tumor tissue were present in tumors expressing mouse endocan (Fig. 6C), or non glycanated mouse and human endocan (Fig. 6E, F) compared to parental cells or control vector-transfected HT-29 tumors (Fig. 6A, B) or human endocan (Fig. 6D).

**Figure 7:**
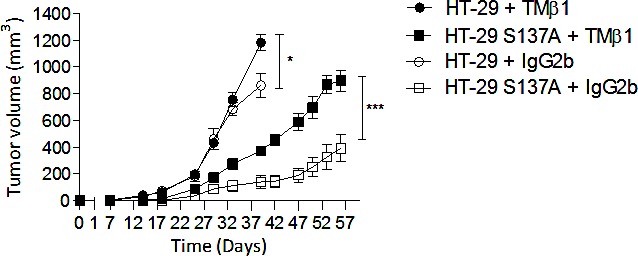
Tumor growth effect of non glycanated human endocan in CD122-depleted mice 400 μg TMβ1 Ab (black color) or isotype control Ab (white color) were i.p. injected 24h before subcutaneously injection of HT-29 cells overexpressing human endocan polypeptide (HT-29 S137A, box), or HT-29 parental (round) (*n*=6 per group) in two independent experiences. *: p<0.05, ***: p<0.001.

In order to study the relationship between this leukocytic infiltration and tumor growth we evaluated the effect of depletion of CD122+ expressing cells (IL-2R-β i.e. NK and NKT cell) on tumor growth by an unique i.p. administration of TMβ1 antibody which results in nearly complete CD122+ cell depletion for 5 weeks, as judged by the absence NKp46+/CD3-cells from the spleen of sentinel mice (data not shown). Administration of the TMβ1 Ab abrogated the anti-tumor effect of the endocan polypeptide while injection of the isotypic control IgG2b Ab had no effect (Fig. 7). The effect of TMβ1 Ab was more pronounced on HT-29 tumors overexpressing the endocan polypeptide than in control tumors. These results suggest that mouse CD122+ cells participate in the tumor growth delay induced by human endocan polypeptide.

## DISCUSSION

Endocan is a chondroitin/dermatan sulfate proteoglycan involved in tumor cell proliferation, endothelial cell migration, and tumor growth [13, 25, 26]. Most of the studies are focused on human endocan, and little is known about the structure and function of mouse endocan. We describe for the first time the structure of mouse endocan, and reveal a novel leukocyte-dependent pathway by which endocan can control the tumor growth.

We show that recombinant and endogenous mouse endocan are much less glycanated than its human ortholog. This lower glycanation rate is consistently observed whatever human or rodent cell types are used for protein production thus excluding a possible cell type dependent effect. Additionally, native mouse endocan either produced by a murine cell line, or precipitated from mouse serum give similar results. This confirms that unlike human endocan, mouse endocan circulates in both glycanated and non glycanated forms. Amino acid sequence and protein conformation are two major regulatory factors proposed to govern the glycanation priorities [27]. It has been previously demonstrated that endocan Δ2, an alternative splicing of human endocan present a variable glycanation [28]. In addition, several studies performed on perlecan and syndecan demonstrate that distant protein domains determine the synthesis of glycosaminoglycan chain [29-31]. Here we show that the N-terminal domains encoded by exons 1 and 2 are involved in determining the glycanation pattern of endocan. Since mouse endocan sequence encoded by exons 1 and 2 differs from human by 12% amino acids, we speculate that combinatorial amino acids sequences within these specific sequences could predict the synthesis of glycosaminoglycan chain.

One critical question that remains to be addressed is whether endocan is actively involved in regulating tumor growth. Several mouse models of tumor xenografts in nude mice using human renal cell carcinoma cells 786-0, human non small lung cancer cells NCI-H1437, or C6 rat glioma cells, demonstrated that endocan overexpression is restricted to the tumor endothelial cells, and notably to the endothelial tip cells [7-10, 32]. These findings raise interesting hypotheses such as if within tumors, the balance between glycanated and non glycanated endocan is shifted in favour of the former or if the tumor microenvironment regulate endocan glycanation at the level of tumor endothelial cells?

Human endocan is known to promote tumor growth [15]. The tumor growth promotion is supported by the fully glycanated endocan through two main mechanisms requiring the presence of a glycan chain: a comitogenic property with several growth factors like FGF-2 or HGF/SF [15, 16], and a chemokinetic property on endothelial cells [11]. In another hand, human endocan was demonstrated to bind *in vitro* onto the leukocyte integrin LFA-1, and thus to inhibit its interaction with the adhesion molecule ICAM-1 [17]. Our present results suggest that this pathway might be involved in endocan's tumor promoting activity through a regulation of leukocyte infiltration into the tumor. Taken together, tumor endothelial cell-derived endocan by its role in regulating endothelial cell migration, tumor cell proliferation, and stromal inflammatory reaction represents one key player in tumor development.

The precise molecular mechanism by which the endocan polypeptide exerts its effect remains to be determined. As a ligand for the leukocyte integrin LFA-1 [33], endocan acts as an inhibitor of LFA-1 / ICAM-1 interaction, an important step in the firm adhesion of leukocytes to the endothelium, and thereby could regulate the leukocyte migration into tumor tissues. Here we showed that CD122+ leukocytes, which represent NK cells in SCID mice, are involved in the endocan polypeptide effect of delaying tumor growth.

Our present findings also suggest that the SCID mouse model of tumor xenograft represents a validated model to explore the mechanism of action of endocan polypeptide in the control of tumor growth, and might open a new way to fight cancer.

## EXPERIMENTAL PROCEDURES

### Cell culture

Human embryonic kidney 293 cell line (HEK293), human colon adenocarcima cell line (HT-29), human lung adenocarcinoma cell line (A549), mouse lymphoma cell line (EL-4), mouse embryonic fibroblast cell line (NIH3T3), human FGF-2-transfected NIH3T3 (K1000), and mouse melanoma cell line (B16F10) were cultured in DMEM (Gibco, USA) containing 10% FCS and 2 mM L-glutamine. The Chinese Hamster Ovarian cell line (CHO-DG44) was cultured in α-MEM (Gibco) supplemented with 10% FCS, HT-supplement and 2 mM L-glutamine.

### Expression of human and mouse endocan

Transient transfections of human and mouse endocan were performed in HEK293, HT-29, A549, CHO-DG44, B16F10 and NIH3T3 cells using FuGENE-6 transfection reagent (Promega, USA). The cell supernatants were recovered 48h following transfection, centrifuged, filtered and stored at −20°C until immunoprecipitation.. The non glycanated mouse endocan/S138A, the mouse endocan/S136M, and the human endocan/M135S were engineered by PCR with the Quick-Change site directed mutagenesis kit, according to the manufacturer's recommendations (Agilent technologies, USA). Different chimaeras constructions were obtained by PCR using *pfu* polymerase and verified by Sanger sequencing. Stably transfected HEK293 cell clones overexpressing wild type human or mouse endocan and non glycanated human endocan/S137A were established by transfection and selection with G418 (350 μg/mL) (Gibco) and sub-cloning as described previously [6]. Recombinant mouse endocan and human endocan/S137A were produced in serum free 293-SFM medium (Gibco). HT-29, K1000, and A549 cells overexpressing mouse or human endocan (wild-tye-WT or mutants) were also established through similar transfection, selection, and cloning procedures. The cell clones were characterized by their specific production rate of endocan measured in ng/24h/10^6^ cells.

### Ion exchange chromatography

The HEK293 cell supernatant was passed through a 0.2 cm x 1.3 cm diethylaminoethyl DEAE- or a Q-Sepharose column (Biorad, France) run originally in 20 mM Tris HCl pH 7.4, containing 0.05 M NaCl. Bound endocan was eluted from the DEAE-Sepharose with 20 mM Tris HCL pH 7.4, containing 1 M NaCl, and then concentrated in a 0.5 ml Vivaspin concentrator with a 30 kD molecular weight cut-off (Vivascience, Germany). Alternatively, bound endocan was eluted with 20 mM Tris HCl pH 7.4, containing a NaCl step gradient from 0.1 to 1 M. Endocan from each elution fraction was measured by ELISA and subjected to western blot analysis [34].

### Chondroitinase ABC

The DEAE-eluted endocan was treated with 1 unit/ml of chondroitinase ABC (Sigma, USA) overnight at 37°C and subjected to western blot analysis.

### Immunoprecipitation of endocan

The anti-endocan mAb MEP14 (Lunginnov, France) was coupled to agarose beads using the Affi-Gel Hz immunoaffinity kit (Bio-Rad, USA). Then, MEP14-agarose beads were added to the cell supernatants or the mouse sera and incubated overnight at 4°C under rolling. Agarose beads were centrifuged, washed 3 times with PBS containing 0.5% NP40 and Complete® protease inhibitor cocktail (Roche, Germany), washed 3 more times with PBS plus protease inhibitor, resuspended in 50 μL SDS-PAGE sample buffer, and immunoblotted. In some samples, 0.1 M DTT were added before western blot.

### Mouse endocan ELISA

Mouse endocan was quantified by ELISA using the following monoclonal antibodies: MEP14 (5 ng/μL) for capture the GG237 (1 μg/mL) for detection as previously described [34].

### Western blot

Polypeptides were separated on a 15% reduced SDS-PAGE gel and blotted onto nitrocellulose membranes (Hybond ECL, Amersham, Germany). After the blocking step, membranes were incubated with HRP-conjugated MEP14 mAb diluted at 1/10,000 (Lunginnov, France) and revealed using the ECL detection kit (Pierce, USA). All incubations were done for 1 hour at room temperature under continuous shaking.

### Purification of human endocan

Recombinant human endocan/S137A, were produced in serum free 293-SFM medium (Gibco). Anti-proteases were added to the cell supernatants, cleared by filtration (0.2 μm), and diluted 1:1 v/v with ultrapure water. A first enrichment step was made by the passage of supernatants through a 2 × 5 cm anion-exchange chromatography column (Capto Q GE Healthcare), washed with 20 mM Tris pH 8, and eluted with a NaCl gradient from 0 to 1 M NaCl in Tris buffer. The eluted fractions were evaluated for the presence of endocan by ELISA and then pooled for affinity chromatography as previously described [6]. Purity of Human endocan/S137A was verified by SDS PAGE silver staining.

### Cell proliferation assays

Cell growth and survival were measured by BrDU incorporation (Roche) and MTT reduction. HT-29 cells were seeded at a density of 0.5 × 10^4^/well in 96-well microplates and cultured for 24 hours in complete medium, including 10% FCS. After 24 hours of starvation in medium without FCS, purified recombinant endocan (glycanated human endocan, endocan/S137A, glycanated mouse endocan, endocan/S138A) was added at various concentrations ranging from 1 to 1000 ng/mL. After 24 hours of culture, BrDU incorporation and MTT viability assay were performed as recommended by manufacturer. Mitomycine (100 ng/mL) was used as control.

### *In vivo* tumor models

Animal experiments were performed as described previously [15]. Briefly, CB-17 scid/scid homozygous mice (male, 5-6 weeks of age) were injected s.c into the right lateral flank. Mice received 10^6^ HEK293 cells, 0.25 × 10^6^ HT-29 cells, 10^6^ A549 cells, 10^5^ K1000 cells resuspended in 200 μl PBS without FCS. Tumor size was assessed once a week and the animals were killed when the largest tumor diameter reached 1 cm. Animals were handled according to the ethical principles of animal experimentation established by the European Center of Tufts University.

Osmotic pumps (reference number 2004 - Alzet, Cupertino, USA) were filled with 200 μL of either purified endocan/S137A or PBS, and then inserted under the dorsal skin of the mice. Tumor HT-29 cell lines were injected at the right lateral flank. For pharmacokinetics studies, blood endocan drawn from retroorbital sinus (100μl/week) and measured at various times points during endocan infusion. For tumor effect study, endocan loaded osmotic pumps were inserted one week before HT-29 tumor cell implantation.

### Pathological analysis

A systematic macroscopic analysis was realized for each organ and the tumor, was disected, fixed in AFA (LaboNord, Templemars, France) and processed for paraffin embedding. Three μm thick paraffin slices were stained with hematoxilin eosin.

### *In vivo* depletion of CD122+ cells

The hybridoma producing the anti-mouse CD122 rat IgG2b monoclonal Ab (TMβ1) was a generous gift from Dr Toshiyuki Tanaka (Osaka University, Japan). The isotype control rat IgG2b monoclonal Ab was a generous gift from Dr Jamal Khalife (Pasteur Institute of Lille). The Ab were produced and purified in house on protein G-sepharose. 400 μg TMβ1 or control IgG2b in PBS were injected i.p. 24h before HT-29 cell injection. The efficiency of the depletion was checked through the presence of NKp46+/CD3- in spleen for sentinel mice at day 1, 19 and 42. Mouse exhibiting a percentage < 2% was considered depleted.
